# Impact of a Nutrient-Dense Oral Nutritional Supplement on Growth in At-Risk Filipino Children: A Single-Arm Study

**DOI:** 10.7759/cureus.113728

**Published:** 2026-07-31

**Authors:** Melchor Victor G Frias, Jossie M Rogacion, Garner A De Los Santos, Jimmy M Bautista, Reinachell D Daclan, Cielo I Ruanto, Cynthia G Wagayen, Grace H Diaz, Mark Anthony A Lansangan

**Affiliations:** 1 Pediatrics, De La Salle Medical and Health Sciences Institute, Dasmariñas, PHL; 2 Pediatrics, San Pedro Calungsod Medical Center, Kawit, PHL; 3 Medical and Scientific Affairs, Nestlé Health Science, Makati City, PHL

**Keywords:** children, malnutrition, nutrient-dense oral supplement, pediatrics, philippines

## Abstract

Growth faltering and dietary inadequacy remain important concerns among Filipino preschool children at nutritional risk. This prospective, community-based, single-arm study evaluated anthropometric and dietary changes during a 120-day intervention of a nutrient-dense oral nutritional supplement (ONS) and dietary counseling.

Children aged three to five years received two 250 mL servings daily (500 kcal/day) of a vanilla-flavored ONS for 120 days. Primary outcomes were changes in weight, height, weight-for-age (WFA), height-for-age (HFA), and weight-for-height (WFH) percentiles and z-scores; secondary outcomes included nutrient adequacy relative to the Philippine Dietary Reference Intake estimated average requirements (EARs), dietary diversity score (DDS), compliance, and health events. Of 268 screened children, 245 were enrolled; 223 completed follow-up and were included in the per-protocol (PP) analysis, and 239 were included in the intention-to-treat (ITT) sensitivity analysis.

In the PP analysis, mean weight increased from 13.1 ± 1.4 kg to 14.2 ± 1.8 kg, and mean height increased from 95.5 ± 5.6 cm to 98.5 ± 5.4 cm (both p < 0.001). Median WFA z-score improved from -1.6 (IQR, -2.1 to -1.2) to -1.3 (IQR, -1.9 to -0.8), HFA z-score from -1.7 (IQR, -2.4 to -0.9) to -1.5 (IQR, -2.3 to -0.8), and WFH z-score from -0.8 (IQR, -1.1 to -0.7) to -0.7 (IQR, -1.1 to -0.3; all p < 0.001). Significant improvements in growth parameters were observed from day 30 and remained significant over the study follow-up. The proportion of children at or below the 25th percentile for WFH decreased from 100% at baseline to 46.2% by day 120. Energy inadequacy decreased from 76.0%-79.4% at baseline to 11.9%-15.6% at day 120, and protein inadequacy decreased from 24.0%-27.8% to 0%-0.8%. Compliance with video-observed treatment (VOT) ranged from 92.5% to 98.3%, and no ONS-related adverse events were recorded.

In conclusion, nutrient-dense ONS combined with dietary counseling was associated with improved anthropometric status and nutrient adequacy over 120 days.

## Introduction

Malnutrition, predominantly undernutrition, is highly prevalent in early childhood. In 2024, the global prevalence of stunting was 23.2% among children under five (over 150 million), while wasting affected 6.6% (45 million) [1]. Asia accounts for most of the global burden of stunting (52%) and wasting (70%) [2], and 20%-45% of children under five are at moderate-to-high nutrition risk [3-5]. In the Philippines, undernutrition is a major public health issue: in 2023, 23.6% of children under five were stunted, placing the country among the top 10 worldwide for stunting [6]. Nearly 26% of Filipino children aged one to two years also suffer from micronutrient undernutrition [7].

Malnutrition drives growth faltering and developmental impairments, limiting functional capacity, cognitive skills, and psychosocial outcomes [8-10]. Moderate-to-high nutrition risk is associated with impaired immunity and a higher risk of recurrent infections, such as pneumonia and diarrhea, which exacerbate nutrient deficiencies and create a vicious cycle of malnutrition and illness [11]. Undernutrition is estimated to contribute to half of all deaths in children under five [2].

Oral nutritional supplements (ONS) are an established intervention for children who are malnourished or unable to meet nutrient needs through diet alone. Clinical trials show that ONS can improve weight gain and reduce recurrent infections in children at nutritional risk [12-14]. However, the composition of ONS significantly impacts its effectiveness in nutritionally at-risk children [15-17]. Polymeric formulas providing a blend of micro- and macronutrients are recommended to address multiple deficiencies, and several nutrients within these formulas have been shown to influence growth [18]. Nutrient-dense ONS have been associated with significant increases in weight-for-height (WFH) and height-for-age (HFA) among nutritionally at-risk children, typically defined as WFH between the 5th and 25th percentiles [12,19], but data on broader nutritional and clinical outcomes remain limited.

In this community-based, single-arm study, longitudinal changes in anthropometric status and nutrient inadequacy were evaluated during a 120-day intervention comprising nutrient-dense ONS and dietary counseling among nutritionally at-risk Filipino preschool children aged three to five years.

## Materials and methods

Study design

This study was a prospective, community-based, single-arm interventional trial that was conducted in Dasmariñas City, Cavite, Philippines, from April to November 2023. The study was conducted in an urban setting and targeted nutritionally at-risk children aged three to five years living in households within randomly selected communities or barangays (an administrative unit of 50-100 families). A list of all barangays within the city was compiled, and study sites were selected using random sampling. Eligible children were enrolled from the selected barangays. All study procedures were conducted in accordance with the latest version of the Declaration of Helsinki and applicable regulatory requirements. The study was approved by the independent ethics committee of the De La Salle Medical and Health Sciences Institute (Ref. No. 2020-30-02-A; date of approval: November 20, 2020). Written informed consent was obtained from all parents or legal guardians before any study procedures. The study protocol was registered at the Philippine FDA’s Center for Food Regulation and Research (No. CFRR-20220614154712; date of registration: September 2, 2022). 

Study participants

The study recruited children aged >36 to 60 months via convenience sampling who were classified as nutritionally at risk. Nutritional risk was defined as having a WFH percentile between the 5th and 25th percentiles according to the World Health Organization (WHO) growth standards [20]. The eligibility criteria were limited to children who had at least one responsible caregiver who could receive instructions and counseling. Preference was given to families where the child was the only one under five years of age to minimize the likelihood of sharing the ONS. Children were excluded from the study if they had any diagnosed chronic illnesses, such as cardiac, pulmonary, neurologic, gastrointestinal, metabolic, or renal conditions, or developmental disabilities. We also excluded children with a documented allergy to cow's milk, those with acute illnesses with or without fever (≥38°C) at the time of recruitment or within one week prior, those who were receiving an ONS at the time of recruitment, and children who were born with low birth weight or small for gestational age (<2500 g).

Study product

The intervention used a dietary formula for children ≥1 year at risk of malnutrition (Nutren Junior®, Nestlé Health Science, Vevey, Switzerland). The vanilla-flavored ONS was nutritionally complete, packaged in 400 g tins, and labeled for the study. The prescribed dose was two servings daily for 120 days; each serving provided 250 mL and 250 kcal, corresponding to a total prescribed intake of 500 kcal/day and 240 servings over the intervention period. The nutrient composition as consumed is summarized in Table 1. All participants received a single dose of mebendazole 500 mg before the first ONS intake unless anthelmintic treatment had been used within the preceding four weeks. Participants were supplied with a one-week quantity of milk and weekly refills thereafter. Parents or caregivers were instructed on ONS preparation and administration, and weekly dietary records were collected during visits.

**Table 1:** Nutrient composition of the oral nutritional supplement per 100 kcal as consumed. Values are presented per 100 kcal of the prepared oral nutritional supplement as consumed. One prepared serving provided 250 mL and 250 kcal. The prescribed daily dose was two servings, equivalent to 500 kcal/day. Ca, calcium; C18:2 n-6, linoleic acid; C18:3 n-3, α-linolenic acid; C22:6 n-3 DHA, docosahexaenoic acid; Cl, chloride; Cr, chromium; Cu, copper; DHA, docosahexaenoic acid; DP, degree of polymerization; Fe, iron; kcal, kilocalorie; kJ, kilojoule; K, potassium; L-carnitine, levocarnitine; MCT, medium-chain triglycerides; Mg, magnesium; mg, milligram; mgTE, milligram α-tocopherol equivalent; Mn, manganese; Mo, molybdenum; MUFA, monounsaturated fatty acids; Na, sodium; P, phosphorus; PUFA, polyunsaturated fatty acids; Se, selenium; SFA, saturated fatty acids; TFA, trans-fatty acids; UoM, unit of measurement; Vit, vitamin; μg, microgram; μgD, microgram vitamin D equivalent; μgRE, microgram retinol equivalent; Zn, zinc

Parameter	UoM	Per 100 kcal (as consumed)
			Energy	kcal	100
Energy	kJ	418.4
Water	g	81.61
Protein	g	3.08
Fat	g	4.4
C18:2 n-6 Linoleic Acid	g	0.53
C18:3 n-3 a-Linolenic Acid	g	0.11
C22:6 n-3 DHA	mg	4.25
Sum MUFA	g	2.23
Sum PUFA	g	0.701083
Sum SFA	g	1.06
Sum TFA	g	0.05
MCT Medium Chain Triglycerides	g	0.81
Carbohydrates	g	12.02
Sum of Fibers ≥DP3	g	0.53
Lactose	g	0.11
Ca (Calcium)	mg	91.35
Mg (Magnesium)	mg	14.87
P (Phosphorus)	mg	70.11
Na (Sodium)	mg	44.61
K (Potassium)	mg	116.84
Cl (Chloride)	mg	80.73
Mn (Manganese)	µg	103.03
Fe (Iron)	mg	1.27
Cu (Copper)	mg	0.06
Zn (Zinc)	mg	0.7
Se (Selenium)	µg	4.31
I (Iodine)	µg	7.01
Cr (Chromium)	µg	1.69
Mo (Molybdenum)	µg	4.48
Vit A	µgRE	44.61
Total Vitamin D [Sum of Vitamin D2+D3]	µgD	1.49
Vit E	mgTE	1.02
Vit K1 (Phytomenadione)	µg	5.31
Vit C	mg	7.01
Vit B1	mg	0.11
Vit B2	mg	0.11
Niacin	mg	0.46
Niacin Equivalent	mg	1.08
Vit B6	mg	0.15
Total Folic Acid	µg	14.44
Pantothenic Acid	mg	0.63
Vit B12	µg	0.21
Biotin	µg	1.81
Choline	mg	28.89
Inositol	mg	5.94
L-Carnitine	mg	6.16

ONS preparation involved handwashing and mixing seven level scoops of powder with 210 mL of cooled boiled water to yield 250 mL (250 kcal) per serving. Children were instructed to consume the milk immediately. Compliance was monitored using video-observed treatment (VOT) and weekly investigational product accountability. For VOT, caregivers submitted videos of preparation and complete intake for both prescribed daily servings through a smartphone messaging application. One to two trained community health workers and a social worker reviewed the videos, provided reminders, clarified preparation or intake issues, and escalated concerns to the investigators. Out of 245 eligible households (4.9%), 12 did not have a smartphone and used devices borrowed from relatives or friends. Empty tins were returned weekly to the assigned community health workers and were reconciled against dispensing logs by the study pharmacist.

A registered dietitian provided dietary counseling throughout the study using the Pinggang Pinoy (Pilipino Plate) model. Counseling focused on age-appropriate feeding practices, including meal frequency, portion size, adequacy of intake, feeding environment, and fostering a positive caregiver-child feeding relationship. Sessions, reinforced at each visit by the dietitian and pediatricians, were tailored to each child’s needs.

Study procedures

Upon obtaining informed consent from the parent or caregiver, demographic data, including the child's date of birth and sex, were collected. A brief medical history was obtained, and physical examination was performed by a pediatrician or study investigator. Anthropometric measurements included weight, height, weight-for-age (WFA), HFA, and WFH. Weight was measured using a standard scale with 0.1-kg precision. Standing height was measured by a pediatrician, study coordinator, or nurse using a height scale and recorded to the nearest 0.1 cm. These measures were compared with WHO growth standards to determine WFA, HFA, and WFH percentiles and z-scores.

A dietitian interviewed parents or caregivers about feeding practices and instructed them on completing a study-specific dietary record. Baseline dietary intake was assessed over two non-consecutive days, at least two weeks apart, to estimate usual intake.

Participants were reassessed at home on days 14, 30, 60, 90, and 120, with a ±5-day visit window. Follow-up assessments included anthropometry, VOT-based compliance assessment, product accountability review, dietary intake review, and adverse-event/health-event monitoring. Caregivers were queried regarding upper respiratory infection, diarrhea, hospitalization, and other infections or disorders. Reported events were assessed for severity, seriousness, causality, ONS action taken, and outcome. Parents or caregivers recorded dietary intake throughout follow-up. A dietitian analyzed nutrient adequacy for energy, carbohydrate, protein, fat, iron, calcium, zinc, and vitamins A, C, and B1 using the Philippine Dietary Reference Intakes. Group nutrient adequacy was summarized as the number and percentage of children meeting or falling below the estimated average requirement (EAR), rather than as percentage recommended dietary allowance. Dietary diversity was evaluated using Food and Agriculture Organization (FAO) individual dietary diversity principles across nine food groups [21]. Because the validated Minimum Dietary Diversity indicator is primarily intended for younger children, dietary diversity score (DDS) was interpreted as an exploratory proxy of dietary variety in this three- to five-year age group.

Sample size and statistical analysis

The sample size for this single-arm study was calculated based on the baseline WFH percentile of 15.9 ± 7.29 reported by Huynh et al. [19]. Assuming a two-sided significance level (alpha) of 0.05 and a 90% statistical power (z_1-β_ = 1.28), the study was powered to detect a 10% relative increase from baseline (corresponding to an absolute increase of 1.59 percentile points, targeting a post-intervention mean of 17.49). The following standard formula for a single-arm mean was applied: $N = \frac{SD^2 \times \left(z_{1-\alpha/2} + z_{1-\beta}\right)^2}{d^2}$, where the standard deviation (SD) was 7.29, and the target difference (d) was 1.59. This yielded a required sample size of 221 participants. To account for an expected attrition rate of 10% due to drop-outs, loss to follow-up, or withdrawals, the final sample size was increased to 245 participants to ensure adequate power.

Descriptive statistics were used to summarize baseline characteristics. Continuous variables were summarized as mean and SD when approximately normally distributed and as median (IQR) when skewed. Categorical variables were summarized as frequency and percentage. The primary analysis used the per-protocol (PP) dataset, defined as participants with evaluable data through day 120. An intention-to-treat (ITT) sensitivity analysis included all enrolled participants who consumed at least one ONS serving. Longitudinal continuous outcomes were analyzed using repeated-measures ANOVA or Friedman's test, according to data distribution. Because several anthropometric percentile and percentage-change outcomes were non-normally distributed and showed an early response followed by stabilization rather than a strictly linear monotonic pattern, the overall repeated-measures test was considered the primary longitudinal analysis. Within-participant comparisons between baseline and post-baseline time points were analyzed using paired t-tests or Wilcoxon signed-rank tests, as appropriate. Pairwise post-baseline testing was adjusted using Bonferroni correction for five comparisons against baseline (adjusted alpha = 0.01). Effect sizes were reported as partial eta-squared for repeated-measures ANOVA, Kendall's W for Friedman tests, and rank-biserial correlation for Wilcoxon signed-rank comparisons. All statistical tests were two-sided.

## Results

**Figure 1:**
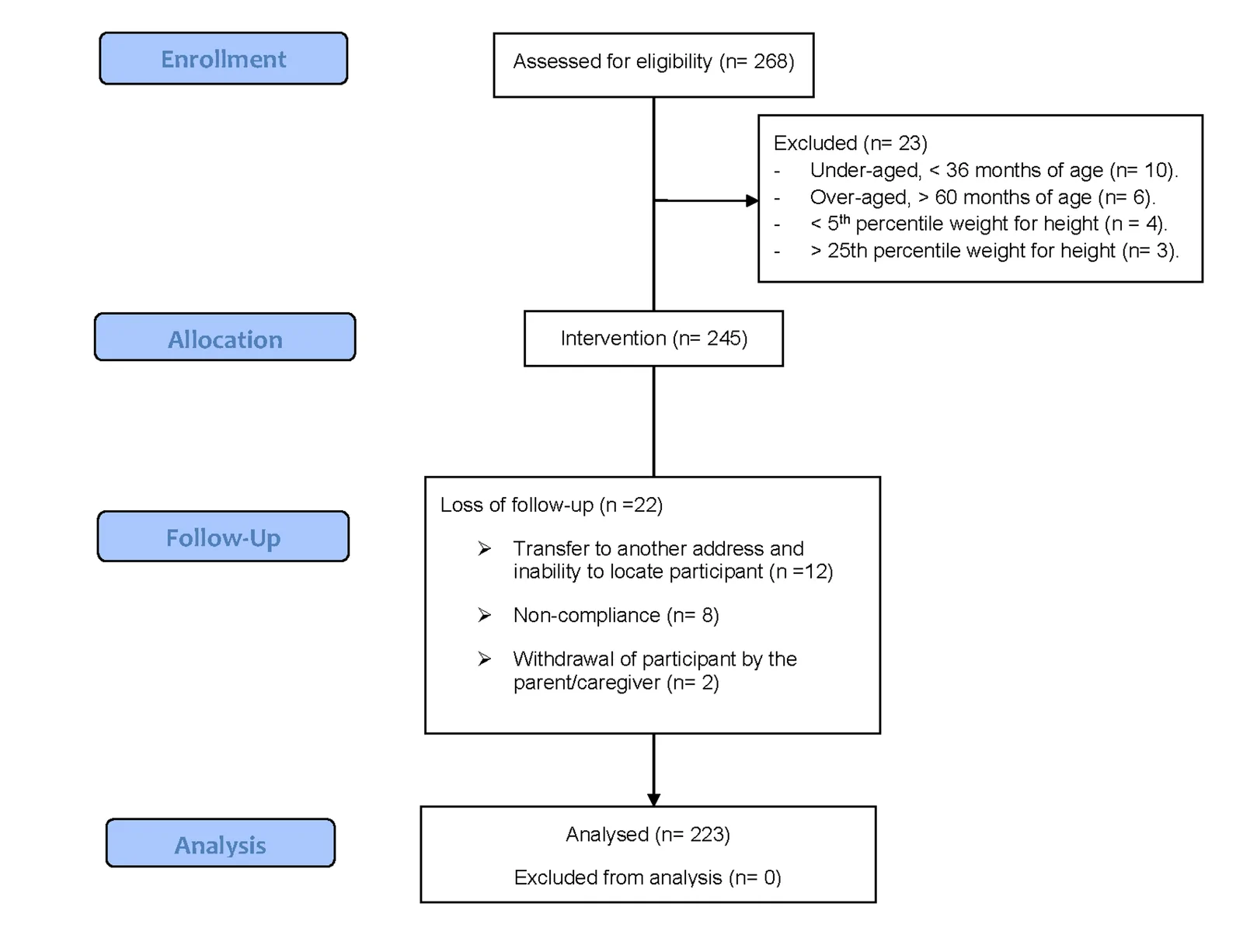
Study's flowchart.

A total of 268 children were screened for eligibility. Twenty-three children (8.6%) were screen failures: 10 were younger than 36 months, six were older than 60 months, four had WFH below the fifth percentile, and three had WFH above the 25th percentile. Overall, 245 children met the eligibility criteria and were enrolled. By the end of follow-up, 223 participants completed the intervention and were included in the PP analysis (attrition, 22/245 (9.0%)). The 22 non-completers included 12 participants who transferred address and could not be located, eight participants who withdrew because of non-compliance or inability to consume the ONS for four consecutive days, and two participants who withdrew by the parent or caregiver because the visit schedule could not be met. Sixteen non-completers had consumed at least one ONS serving and were included in the ITT sensitivity analysis (n = 239; Figure 1).

In the PP dataset, the mean age was 4 ± 0.6 years, and 127 participants (57.0%) were female. Most primary caregivers were mothers (85.7%), as shown in Table 2. Overall, mean baseline weight was 13.1 ± 1.4 kg, mean height was 95.5 ± 5.6 cm, mean WFA percentile was 9.1 ± 11.1, mean HFA percentile was 12.9 ± 18.7, and mean WFH percentile was 18.0 ± 6.2. The average daily caloric intake was 1,060.8 ± 336.1 kcal among participants with dietary records (Table 2).

**Table 2:** Demographic and household characteristics of the study population. PHP, Philippine peso; SD, standard deviation

Characteristic	Level	Overall (N = 223)
Child characteristics		
Age, years	Mean (SD)	4.0 (0.6)
Sex, n (%)	Male	96 (43.0%)
	Female	127 (57.0%)
Household characteristics		
Family Income (PHP)	Mean (SD)	9,215.3 (5,771.0)
Average Number of Household Members	Mean (SD)	4.5 (1.2)
Maternal educational attainment, n (%)	Elementary undergraduate	14 (6.3%)
	Elementary graduate	37 (16.6%)
	High-school undergraduate	27 (12.1%)
	High-school graduate	106 (47.5%)
	College undergraduate	21 (9.4%)
	College graduate	11 (4.9%)
	Vocational education	7 (3.1%)
Paternal educational attainment, n (%)	Elementary undergraduate	11 (4.9%)
	Elementary graduate	44 (19.7%)
	High-school undergraduate	15 (6.7%)
	High-school graduate	99 (44.4%)
	College undergraduate	34 (15.3%)
	College graduate	18 (8.1%)
	Vocational education	2 (0.9%)
Primary caregiver, n (%)	Mother	191 (85.7%)
	Father	6 (2.7%)
	Grandmother/grandparent	14 (6.3%)

Baseline anthropometric measures were generally comparable between male and female participants, with similar mean height, weight, WFA percentile, and WFH percentile values. Females had a numerically higher HFA percentile and a higher daily caloric intake than males (Table 3).

**Table 3:** Baseline anthropometric characteristics by sex in the per-protocol dataset. *Dietary records were available for 222 participants overall. PHP, Philippine peso; SD, standard deviation; WFA, weight-for-age; HFA, height-for-age; WFH, weight-for-height

Characteristic	Male (n = 96)	Female (n = 127)	Overall (n = 223)
Height, cm	95.1 (5.5)	95.9 (5.6)	95.5 (5.6)
Weight, kg	13.1 (1.4)	13.1 (1.4)	13.1 (1.4)
WFA percentile	8.2 (11.0)	9.9 (11.2)	9.1 (11.1)
HFA percentile	10.7 (17.0)	14.5 (19.8)	12.9 (18.7)
WFH percentile	17.8 (6.6)	18.2 (5.9)	18.0 (6.2)
Daily caloric intake, kcal	998.7 (322.7)	1,142.3 (337.5)*	1,060.8 (336.1)*

Changes in anthropometric measurements

In the PP analysis, absolute growth increased significantly during follow-up. Mean height increased from 95.5 ± 5.6 cm to 98.5 ± 5.4 cm (partial eta-squared = 0.799; p < 0.001), corresponding to a median height change of 3.0% (IQR, 2.0%-4.0%) by day 120. Mean weight increased from 13.1 ± 1.4 kg at day 0 to 14.2 ± 1.6 kg at day 120 (partial eta-squared = 0.476; p < 0.001). Median percentage weight change reached 8.0% (IQR, 5.0%-11.0%) by day 120 (Kendall's W = 0.303; p < 0.001; Table 4). A measurable early growth response was already observed by day 30, when mean height had increased to 96.7 ± 5.5 cm and mean weight had increased to 13.8 ± 1.6 kg; median percentage changes at day 30 were 1.0% (IQR, 1.0%-2.0%) for height and 5.0% (IQR, 2.0%-8.0%) for weight.

**Table 4:** Weight and height and % change (per-protocol dataset). Overall effect sizes are partial eta-squared for repeated-measures ANOVA and Kendall's W for Friedman tests. Pairwise post-baseline comparisons used Wilcoxon signed-rank tests with Bonferroni correction (adjusted alpha = 0.01). IQR, interquartile range; SD, standard deviation; ANOVA, analysis of variance

Variables	Day 0	Day 15	Day 30	Day 60	Day 90	Day 120	Test statistic	Effect size	p-value
Height (cm) (Mean ± SD)	95.5 ± 5.6	96.2 ± 5.5	96.7 ± 5.5	97.4 ± 5.4	97.9 ± 5.4	98.5 ± 5.4	1877.385	0.799	< 0.001
% Change in Height, Median (IQR)	-	1.0 (0.0, 1.0)	1.0 (1.0, 2.0)	1.0 (1.0, 2.0)	2.0 (1.0, 3.0)	3.0 (2.0, 4.0)	820.705	0.789	< 0.001
Weight (kg) (Mean ± SD)	13.1 ± 1.4	13.6 ± 1.5	13.8 ± 1.6	13.9 ± 1.6	14.1 ± 1.7	14.2 ± 1.8	201.418	0.476	< 0.001
% Change in Weight, Median (IQR)	-	4.0 (1.0, 7.0)	5.0 (2.0, 8.0)	5.0 (2.0, 8.0)	6.0 (3.0, 9.0)	8.0 (5.0, 11.0)	96.685	0.303	< 0.001

**Figure 2:**
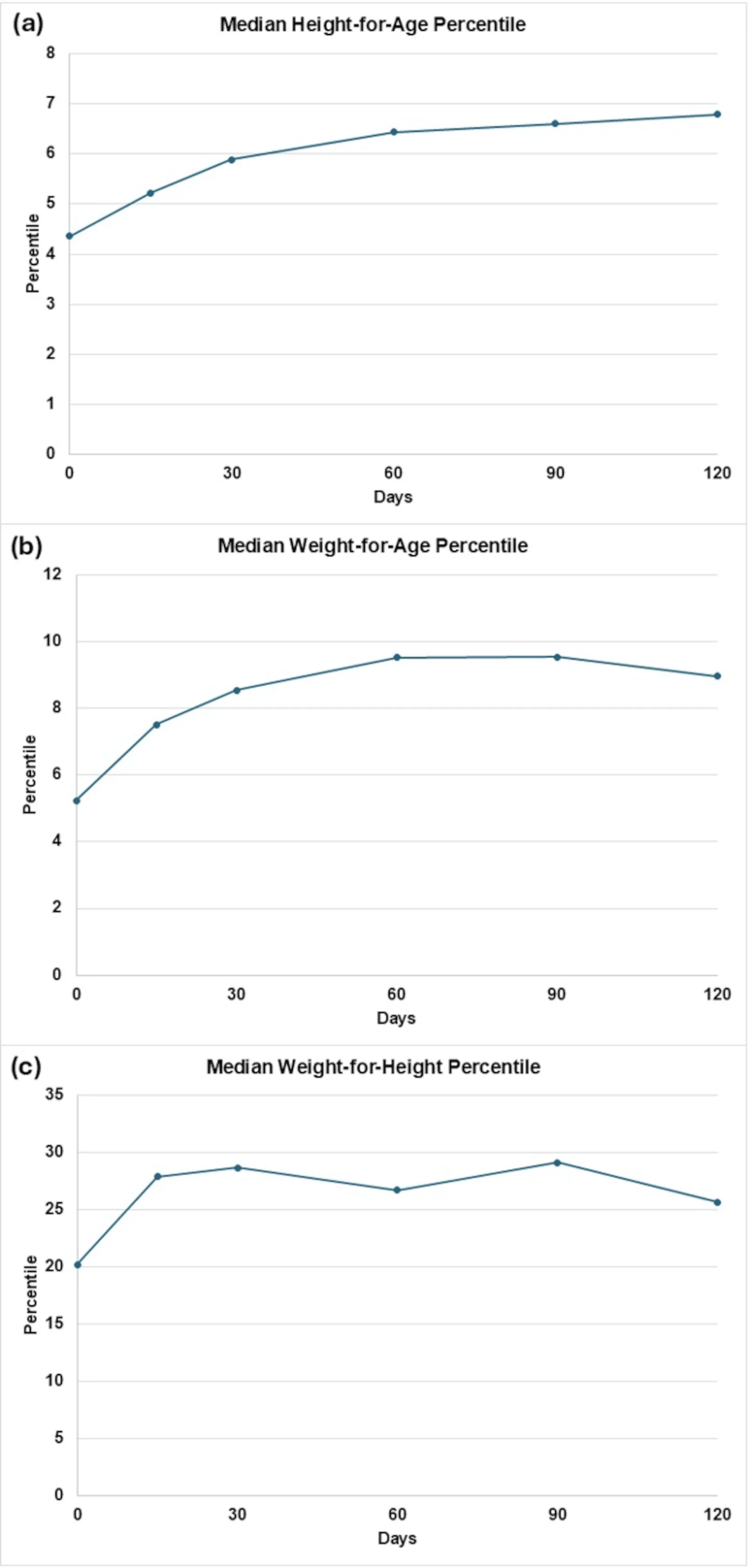
Changes in median anthropometric percentiles over 120 days. (a) Height-for-age; (b) Weight-for-age; (c) Weight-for-height.

Median HFA percentile increased from 4.4 (IQR, 0.9-18.0) at baseline to 6.8 (IQR, 1.2-20.5) at day 120 (Kendall's W = 0.115; p < 0.001; Figure 2a). Median HFA z-score improved from -1.7 (IQR, -2.4 to -0.9) to -1.5 (IQR, -2.3 to -0.8; Kendall's W = 0.122; p < 0.001). The proportion of children at or below the 25th HFA percentile changed from 185 (83.0%) at baseline to 183 (82.1%) at day 120. Pairwise improvement was not statistically significant at day 15 (adjusted p = 0.097) but was significant from day 30 onward (rank-biserial correlation range, 0.339-0.518; adjusted p < 0.001; Table 5).

**Table 5:** Longitudinal changes in height-for-age percentiles and z-scores in the per-protocol dataset. Data are presented as median (IQR) or n (%). Day 0 was considered the baseline assessment. Overall longitudinal changes across visits were assessed using Friedman’s test. Overall effect sizes are reported as partial eta-squared for repeated-measures ANOVA and Kendall’s W for Friedman tests, as applicable. Pairwise post-baseline comparisons were performed against day 0 using Wilcoxon signed-rank tests with Bonferroni correction; the adjusted alpha level was 0.01. Pairwise effect sizes are reported where applicable. **Pairwise test statistic, effect size, and p-value for comparison with day 0. HFA, height-for-age; IQR, interquartile range; ANOVA, analysis of variance

Variables		Day 0	Day 15	Day 30	Day 60	Day 90	Day 120	Test statistic	Effect size	p-value
HFA median (IQR)		4.4 (0.9, 18.0)	5.2 (1.1, 17.7)	5.9 (1.2, 20.6)	6.4 (1.2, 20.7)	6.6 (1.2, 19.2)	6.8 (1.2, 20.5)	28.760	0.115	< 0.001
HFA z-scores median (IQR)		-1.7 (-2.4, -0.9)	-1.6 (-2.3, -0.9)	-1.6 (-2.3, -0.8)	-1.5 (-2.3, -0.8)	-1.5 (-2.3, -0.9)	-1.5 (-2.3, -0.8)	30.872	0.122	< 0.001
% Change in HFA, median (IQR)		-	0.0 (-0.3, 1.3)	0.2 (-0.2, 2.8)	0.2 (-0.2, 2.8)	0.7 (0.0, 2.7)	0.7 (0.0, 2.8)	14.908	0.063	< 0.001
Statistic**		-	1.66	4.35	6.06	6.11	6.67	-		
Effect Size**		-	0.13	0.34	0.47	0.48	0.52			
p-value		-	0.097	< 0.001	< 0.001	< 0.001	< 0.001			
HFA Percentiles, N (%)	≤25th	185 (83.0%)	186 (83.4%)	184 (82.5%)	183 (82.1%)	182 (81.6%)	183 (82.1%)	-	-	-
	26-50th	25 (11.2%)	27 (12.1%)	29 (13.0%)	29 (13.0%)	29 (13.0%)	24 (10.8%)			
	51-75th	8 (3.6%)	6 (2.7%)	7 (3.1%)	8 (3.6%)	9 (4.0%)	14 (6.3%)			
	>75th	5 (2.2%)	4 (1.8%)	3 (1.3%)	3 (1.3%)	3 (1.3%)	2 (0.9%)			

Median WFA percentile increased from 5.2 (IQR, 1.8-12.2) at baseline to 9.0 (IQR, 3.0-21.2) at day 120 (Kendall's W = 0.171; p < 0.001; Figure 2b). Median WFA z-score improved from -1.6 (IQR, -2.1 to -1.2) to -1.3 (IQR, -1.9 to -0.8; Kendall's W = 0.169; p < 0.001). The proportion of children at or below the 25th WFA percentile decreased from 205/223 (91.9%) at baseline to 180/223 (80.7%) at day 120. Pairwise comparisons against baseline were significant at each post-baseline time point (rank-biserial correlation range, 0.690-0.762; all adjusted p < 0.001; Table 6).

**Table 6:** Longitudinal changes in weight-for-age percentiles and z-scores in the per-protocol dataset. Data are presented as median (interquartile range) or n (%). Day 0 was considered the baseline assessment. Overall longitudinal changes across visits were assessed using Friedman’s test. Overall effect sizes are reported as partial eta-squared for repeated-measures ANOVA and Kendall’s W for Friedman tests, as applicable. Pairwise post-baseline comparisons were performed against day 0 using Wilcoxon signed-rank tests with Bonferroni correction; the adjusted alpha level was 0.01. Pairwise effect sizes are reported where applicable. **Pairwise test statistic, effect size, and p-value for comparison with day 0. IQR, interquartile range; WFA, weight-for-age; ANOVA, analysis of variance

Variables		Day 0	Day 15	Day 30	Day 60	Day 90	Day 120	Test statistic	Effect size	p-value
WFA median (IQR)		5.2 (1.8, 12.2)	7.5 (2.9, 16.2)	8.5 (3.3, 18.4)	9.5 (3.2, 19.7)	9.5 (3.0, 22.2)	9.0 (3.0, 21.2)	45.780	0.171	< 0.001
WFA z-scores median (IQR)		-1.6 (-2.1, -1.2)	-1.4 (-1.9, -1.0)	-1.4 (-1.8, -0.9)	-1.3 (-1.9, -0.9)	-1.3 (-1.9, -0.8)	-1.3 (-1.9, -0.8)	45.205	0.169	< 0.001
% Change in WFA, median (IQR)		-	1.9 (0.2, 5.8)	2.4 (0.3, 6.8)	2.4 (0.3, 6.8)	2.5 (0.3, 7.8)	2.0 (0.2, 7.4)	3.787	0.016	0.005
Statistic**		-	9.19	9.85	9.79	9.86	8.92	-		
Effect Size**		-	0.72	0.76	0.75	0.76	0.69			
p-value		-	< 0.001	< 0.001	< 0.001	< 0.001	< 0.001			
WFA Percentiles, N (%)	≤25th	205 (91.9%)	190 (85.2%)	183 (82.1%)	183 (82.1%)	177 (79.4%)	180 (80.7%)	-	-	-
	26-50th	16 (7.2%)	27 (12.1%)	32 (14.3%)	33 (14.8%)	40 (17.9%)	32 (14.3%)			
	51-75th	2 (0.9%)	5 (2.2%)	7 (3.1%)	5 (2.2%)	4 (1.8%)	9 (4.0%)			
	>75th	0 (0.0%)	1 (0.4%)	1 (0.4%)	2 (0.9%)	2 (0.9%)	2 (0.9%)			

Median WFH percentile increased rapidly from 20.3 (IQR, 12.7-22.9) at baseline to 27.9 (IQR, 16.0-37.7) by day 15 and was 25.7 (IQR, 14.5-39.6) at day 120 (Kendall's W = 0.116; p < 0.001; Figure 2c). Median WFH z-score improved from -0.8 (IQR, -1.1 to -0.7) at baseline to -0.7 (IQR, -1.1 to -0.3) at day 120, with the highest median value observed at day 90 (-0.5; IQR, -1.0 to -0.2). The proportion of children at or below the 25th WFH percentile decreased from 223/223 (100%) at baseline to 95/223 (42.6%) at day 15 and 103/223 (46.2%) at day 120. Pairwise comparisons against baseline were significant at all post-baseline time points (rank-biserial correlation range, 0.638-0.771; adjusted p < 0.001; Table 7).

**Table 7:** Longitudinal changes in weight-for-height percentiles and z-scores in the per-protocol dataset. Data are presented as median (IQR) or n (%). Day 0 was considered the baseline assessment. Overall longitudinal changes across visits were assessed using Friedman’s test. Overall effect sizes are reported as partial eta-squared for repeated-measures ANOVA and Kendall’s W for Friedman tests, as applicable. Pairwise post-baseline comparisons were performed against day 0 using Wilcoxon signed-rank tests with Bonferroni correction; the adjusted alpha level was 0.01. Pairwise effect sizes are reported where applicable. **Pairwise test statistic, effect size, and p-value for comparison with day 0. IQR, interquartile range; WFH, weight-for-height; ANOVA, analysis of variance

Variables		Day 0	Day 15	Day 30	Day 60	Day 90	Day 120	Test statistic	Effect size	p-value
WFHmedian (IQR)		20.3 (12.7, 22.9)	27.9 (16.0, 37.7)	28.7 (15.9, 39.5)	26.8 (15.6, 41.4)	29.1 (17.0, 40.8)	25.7 (14.5, 39.6)	29.245	0.116	< 0.001
WFH z-scores median (IQR)		-0.8 (-1.1, -0.7)	-0.6 (-1.0, -0.3)	-0.6 (-1.0, -0.3)	-0.6 (-1.0, -0.2)	-0.5 (-1.0, -0.2)	-0.7 (-1.1, -0.3)	29.245	0.116	< 0.001
% Change in WFH, median (IQR)		-	9.3 (0.0, 17.6)	9.3 (0.0, 19.2)	9.3 (0.0, 19.2)	8.5 (-0.8, 19.9)	7.7 (-2.0, 19.2)	0.153	0.001	0.962
Statistic**		-	9.53	9.61	9.10	9.98	8.27	-		
Effect Size**		-	0.75	0.75	0.70	0.77	0.64			
p-value		-	< 0.001	< 0.001	< 0.001	< 0.001	< 0.001			
WFH Percentiles, N (%)	≤25th	223 (100%)	95 (42.6%)	96 (43.0%)	105 (47.1%)	98 (43.9%)	103 (46.2%)	-	-	-
	26-50th	0 (0.0%)	103 (46.2%)	97 (43.5%)	92 (41.3%)	94 (42.2%)	93 (41.7%)			
	51-75th	0 (0.0%)	23 (10.3%)	26 (11.7%)	22 (9.9%)	27 (12.1%)	21 (9.4%)			
	>75th	0 (0.0%)	2 (0.9%)	4 (1.8%)	4 (1.8%)	4 (1.8%)	6 (2.7%)			

Changes in nutrient intake and adequacy

Nutrient adequacy was reanalyzed using the EAR pass/fail status based on the Philippine Dietary Reference Intakes. At baseline, energy inadequacy was high in both sexes (female, 79.4%; male, 76.0%-78.1%). By day 15, energy inadequacy had decreased to 18.3% in females and 25.0% in males, and by day 120, it was 11.9% and 15.6%, respectively. Protein inadequacy decreased from 24.0%-27.8% at baseline to 0%-0.8% at day 120. Calcium, iron, vitamin A, and vitamin B1 inadequacy also decreased substantially by day 120. Fat and vitamin C inadequacy improved but remained present in approximately one-quarter to one-third of participants at day 120. Zinc inadequacy remained frequent despite partial improvement, decreasing from 84.9%-88.5% at baseline to 65.1%-68.8% at day 120 (Table 8).

**Table 8:** Range of nutrient inadequacy relative to estimated average requirements. EAR, estimated average requirement

Nutrient	Baseline EAR inadequacy, % range	Day 30, % range	Day 60, % range	Day 90, % range	Day 120, % range
Energy	76.0-79.4	15.9-29.2	11.5-15.1	11.1-11.5	11.9-15.6
Carbohydrate	18.8-23.0	11.1-14.6	12.5-17.5	15.1-15.6	15.1-20.8
Protein	24.0-27.8	0.0-1.0	0.0-2.1	0.0-0.8	0.0-0.8
Fat	37.5-41.3	24.6-31.3	27.1-28.6	31.3-33.3	34.1-35.4
Calcium	77.1-81.0	27.1-31.0	20.6-25.0	13.5-19.0	20.8-24.6
Iron	57.3-67.7	11.9-16.7	7.1-11.5	5.2-7.1	6.3-9.4
Vitamin A	76.0-92.1	6.3-7.1	5.6-6.3	4.2-5.6	3.1-7.9
Vitamin B1	45.8-59.5	0.0-0.0	0.8-1.0	0.0-0.8	2.1-3.2
Vitamin C	41.7-53.1	32.3-38.1	25.4-26.0	24.0-29.4	27.1-31.7
Zinc	84.9-88.5	75.4-77.1	70.6-76.0	64.3-75.0	65.1-68.8

Dietary diversity also improved. Seventy percent of children (154/220) had a higher DDS at the end of follow-up. Median DDS increased from 4 (range, 2-8) at baseline to 5 (range, 3-9) at day 120, and the median percentage change in DDS was 33.3% (range, -50.0% to 250.0%; p < 0.001). Because DDS was adapted for children aged three to five years, it was interpreted as a descriptive indicator of dietary variety rather than as a validated diagnostic measure of micronutrient adequacy.

Intervention adherence and health events

VOT-based compliance ranged from 92.5% to 98.3% of prescribed doses. Participants missed a mean of 6.84 ± 2.42 prescribed intakes out of 240 total prescribed intakes; the median and mode numbers of missed intakes were 6 and 5, respectively. The most commonly reported reasons for missed doses were work or errands (43.9%), household chores (32.3%), and school or daycare (17.0%). Product accountability based on returned tins was complete.

Health events were monitored throughout follow-up. Upper respiratory infections were the most frequently recorded events and increased from 24/223 children (10.76%) at day 15 to 67/213 children (31.46%) at day 120. Diarrhea remained infrequent but increased from 2/223 children (0.90%) at day 15 to 11/213 children (5.16%) at day 120, whereas hospitalization was recorded in four children overall and was not recorded at days 90 or 120 (Table 9). All adverse events were mild to moderate and non-serious unless hospitalization was required. All four serious adverse events met seriousness criteria because of hospitalization; none were judged related to the ONS, no dose modification, interruption, or discontinuation was required, and all affected participants recovered.

**Table 9:** Health events recorded during follow-up. Data are presented as n (%). Percentages were calculated using the number of participants assessed at each follow-up visit as the denominator.

Assessment	N	Upper respiratory infection, n (%)	Diarrhea, n (%)	Hospitalization, n (%)	Other infections/disorders, n (%)
Day 15	223	24 (10.76%)	2 (0.90%)	1 (0.45%)	18 (8.07%)
Day 30	223	26 (11.66%)	2 (0.90%)	1 (0.45%)	24 (10.76%)
Day 60	223	31 (13.90%)	2 (0.90%)	2 (0.90%)	35 (15.70%)
Day 90	220	33 (15.00%)	5 (2.27%)	0 (0.00%)	31 (13.90%)
Day 120	213	67 (31.46%)	11 (5.16%)	0 (0.00%)	21 (9.86%)

ITT sensitivity analysis

The ITT analysis showed findings consistent with the primary PP analysis. Mean height increased from 95.4 ± 5.6 cm at day 0 to 98.3 ± 5.6 cm at day 120 (p < 0.001; effect size, 0.778), and mean weight increased from 13.1 ± 1.4 kg to 14.2 ± 1.8 kg (p < 0.001; effect size, 0.477), as shown in Tables 10-11.

**Table 10:** Longitudinal changes in height, weight, and percentage change (ITT dataset). Data are presented as mean ± standard deviation or median (IQR), as appropriate. Day 0 was considered the baseline assessment. Overall longitudinal changes across visits were assessed using repeated-measures ANOVA for normally distributed variables and Friedman’s test for non-normally distributed variables, as applicable. Overall effect sizes are reported as partial eta-squared for repeated-measures ANOVA and Kendall’s W for Friedman tests. IQR, interquartile range; ITT, intention-to-treat; SD, standard deviation; ANOVA, analysis of variance

Variables	Day 0	Day 15	Day 30	Day 60	Day 90	Day 120	Test statistic	Effect size	p-value
Height (cm) (Mean ± SD)	95.4 ± 5.6	96.1 ± 5.6	96.6 ± 5.6	97.3 ± 5.5	97.8 ± 5.5	98.3 ± 5.6	836.300	0.778	< 0.001
% Change in Height, Median (IQR)	-	1.0 (0.0, 1.0)	1.0 (1.0, 2.0)	1.0 (1.0, 2.0)	2.0 (1.0, 2.0)	3.0 (2.0, 4.0)	781.106	0.766	< 0.001
Weight (kg) (Mean ± SD)	13.1 ± 1.4	13.6 ± 1.6	13.8 ± 1.6	13.9 ± 1.6	14.1 ± 1.7	14.2 ± 1.8	216.900	0.477	< 0.001
% Change in Weight, Median (IQR)	-	4.0 (2.0, 7.0)	5.0 (2.0, 8.0)	5.0 (2.0, 8.0)	6.0 (3.0, 9.0)	8.0 (5.0, 11.0)	94.156	0.283	< 0.001

**Table 11:** HFA z-scores and % change (ITT dataset). Data are presented as median (IQR) or n (%), as appropriate. Day 0 was considered the baseline assessment. Overall longitudinal changes across visits were assessed using Friedman’s test. Overall effect sizes are reported as Kendall’s W. Pairwise post-baseline comparisons were performed against day 0 using Wilcoxon signed-rank tests with Bonferroni correction; the adjusted alpha level was 0.01. Pairwise effect sizes are reported where applicable. **Pairwise test statistic, effect size, and p-value for comparison with day 0. HFA, height-for-age; IQR, interquartile range; ITT, intention-to-treat; ANOVA, analysis of variance

Variables		Day 0	Day 15	Day 30	Day 60	Day 90	Day 120	Test statistic	Effect size	p-value
HFA median (IQR)		3.6 (0.9, 17.9)	4.2 (1.1, 17.6)	5.5 (1.2, 20.4)	5.7 (1.1, 20.5)	5.8 (1.2, 18.7)	5.7 (1.0, 19.9)	24.081	0.092	< 0.001
HFA z-scores median (IQR)		-1.8 (-2.4, -0.9)	-1.7 (-2.3, -0.9)	-1.6 (-2.3, -0.8)	-1.6 (-2.3, -0.8)	-1.6 (-2.3, -0.9)	-1.6 (-2.3, -0.8)	25.816	0.098	< 0.001
% Change in HFA, median (IQR)		-	0.0 (-0.3, 1.2)	0.2 (-0.2, 2.8)	0.2 (-0.2, 2.8)	0.7 (-0.1, 2.7)	0.6 (-0.1, 2.7)	9.627	0.039	< 0.001
Statistic**		-	1.93	4.43	6.08	6.08	6.52	-		
Effect Size**		-	0.15	0.33	0.46	0.46	0.49			
p-value		-	0.054	< 0.001	< 0.001	< 0.001	< 0.001			
HFA Percentiles, N (%)	≤25th	200 (83.7%)	199 (83.3%)	197 (82.4%)	198 (82.8%)	195 (81.6%)	198 (82.8%)	-	-	-
	26-50th	26 (10.9%)	30 (12.6%)	32 (13.4%)	30 (12.6%)	32 (13.4%)	25 (10.5%)			
	51-75th	8 (3.3%)	6 (2.5%)	7 (2.9%)	8 (3.3%)	9 (3.8%)	14 (5.9%)			
	>75th	5 (2.1%)	4 (1.7%)	3 (1.3%)	3 (1.3%)	3 (1.3%)	2 (0.8%)			

Significant longitudinal improvements were also observed for HFA percentile, HFA z-score, WFA percentile, WFA z-score, WFH percentile, and WFH z-score (all p < 0.001), as shown in Tables 12-13. The direction and magnitude of change were comparable to those observed in the PP analysis. In the ITT dataset, the proportion of children at or below the 25th percentile for WFH decreased from 100% at day 0 to 45.6% at day 120, with a marked reduction already observed by day 15 (41.8%). These findings suggest that the observed growth improvements were not materially altered by participant attrition or protocol non-completion. Full ITT results are provided in Tables 10-13.

**Table 12:** WFA z-scores and % change (ITT dataset). Data are presented as median (IQR) or n (%), as appropriate. Day 0 was considered the baseline assessment. Overall longitudinal changes across visits were assessed using Friedman’s test. Overall effect sizes are reported as Kendall’s W. Pairwise post-baseline comparisons were performed against day 0 using Wilcoxon signed-rank tests with Bonferroni correction; the adjusted alpha level was 0.01. Pairwise effect sizes are reported where applicable. **Pairwise test statistic, effect size, and p-value for comparison with day 0. IQR, interquartile range; ITT, intention-to-treat; WFA, weight-for-age

Variables		Day 0	Day 15	Day 30	Day 60	Day 90	Day 120	Test statistic	Effect size	p-value
WFA median (IQR)		5.0 (1.8, 12.2)	7.5 (2.9, 17.1)	8.5 (3.1, 18.3)	9.5 (3.2, 19.4)	9.5 (3.0, 21.7)	8.2 (2.9, 20.5)	49.062	0.171	< 0.001
WFA z-scores median (IQR)		-1.6 (-2.1, -1.2)	-1.4 (-1.9, -0.9)	-1.4 (-1.9, -0.9)	-1.3 (-1.9, -0.9)	-1.3 (-1.9, -0.8)	-1.4 (-1.9, -0.8)	48.532	0.169	< 0.001
% Change in WFA, median (IQR)		-	2.1 (0.2, 5.9)	2.4 (0.3, 7.5)	2.4 (0.3, 7.5)	2.5 (0.4, 8.3)	2.1 (0.2, 7.4)	2.482	0.010	0.042
Statistic**		-	9.80	10.33	10.32	10.32	9.38	-		
Effect Size**		-	0.73	0.77	0.77	0.77	0.70			
p-value		-	< 0.001	< 0.001	< 0.001	< 0.001	< 0.001			
WFA Percentiles, N (%)	≤25th	221 (92.5%)	205 (85.8%)	196 (82.0%)	198 (82.8%)	190 (79.5%)	195 (81.6%)	-	-	-
	26-50th	16 (6.7%)	28 (11.7%)	34 (14.2%)	34 (14.2%)	43 (18.0%)	33 (13.8%)			
	51-75th	2 (0.8%)	5 (2.1%)	8 (3.3%)	5 (2.1%)	4 (1.7%)	9 (3.8%)			
	>75th	0 (0.0%)	1 (0.4%)	1 (0.4%)	2 (0.8%)	2 (0.8%)	2 (0.8%)			

**Table 13:** WFH z-scores and % change (ITT dataset). Data are presented as median (IQR) or n (%), as appropriate. Day 0 was considered the baseline assessment. Overall longitudinal changes across visits were assessed using Friedman’s test. Overall effect sizes are reported as Kendall’s W. Pairwise post-baseline comparisons were performed against day 0 using Wilcoxon signed-rank tests with Bonferroni correction; the adjusted alpha level was 0.01. Pairwise effect sizes are reported where applicable. **Pairwise test statistic, effect size, and p-value for comparison with day 0. IQR, interquartile range; ITT, intention-to-treat; WFH, weight-for-height

Variables		Day 0	Day 15	Day 30	Day 60	Day 90	Day 120	Test statistic	Effect size	p-value
WFH median (IQR)		20.2 (12.0, 22.7)	28.0 (16.0, 38.2)	29.9 (16.1, 39.9)	27.0 (15.6, 41.9)	29.4 (16.8, 42.2)	25.8 (14.5, 40.3)	32.473	0.120	< 0.001
WFH z-scores median (IQR)		-0.8 (-1.2, -0.7)	-0.6 (-1.0, -0.3)	-0.5 (-1.0, -0.3)	-0.6 (-1.0, -0.2)	-0.5 (-1.0, -0.2)	-0.6 (-1.1, -0.2)	32.473	0.120	< 0.001
% Change in WFH, median (IQR)		-	9.7 (0.0, 18.6)	9.8 (0.0, 19.7)	9.8 (0.0, 19.7)	9.0 (-0.5, 21.4)	7.9 (-2.0, 19.7)	0.087	0.00037	0.986
Statistic**		-	10.10	10.01	9.64	10.33	8.69	-		
Effect Size**		-	0.76	0.75	0.72	0.77	0.65			
p-value		-	< 0.001	< 0.001	< 0.001	< 0.001	< 0.001			
WFH Percentiles, N (%)	≤25th	239 (100%)	100 (41.8%)	102 (42.7%)	110 (46.0%)	104 (43.5%)	109 (45.6%)	-	-	-
	26-50th	0 (0.0%)	108 (45.2%)	101 (42.3%)	97 (40.6%)	97 (40.6%)	96 (40.2%)			
	51-75th	0 (0.0%)	29 (12.1%)	32 (13.4%)	28 (11.7%)	34 (14.2%)	28 (11.7%)			
	>75th	0 (0.0%)	2 (0.8%)	4 (1.7%)	4 (1.7%)	4 (1.7%)	6 (2.5%)			

**Figure 3:**
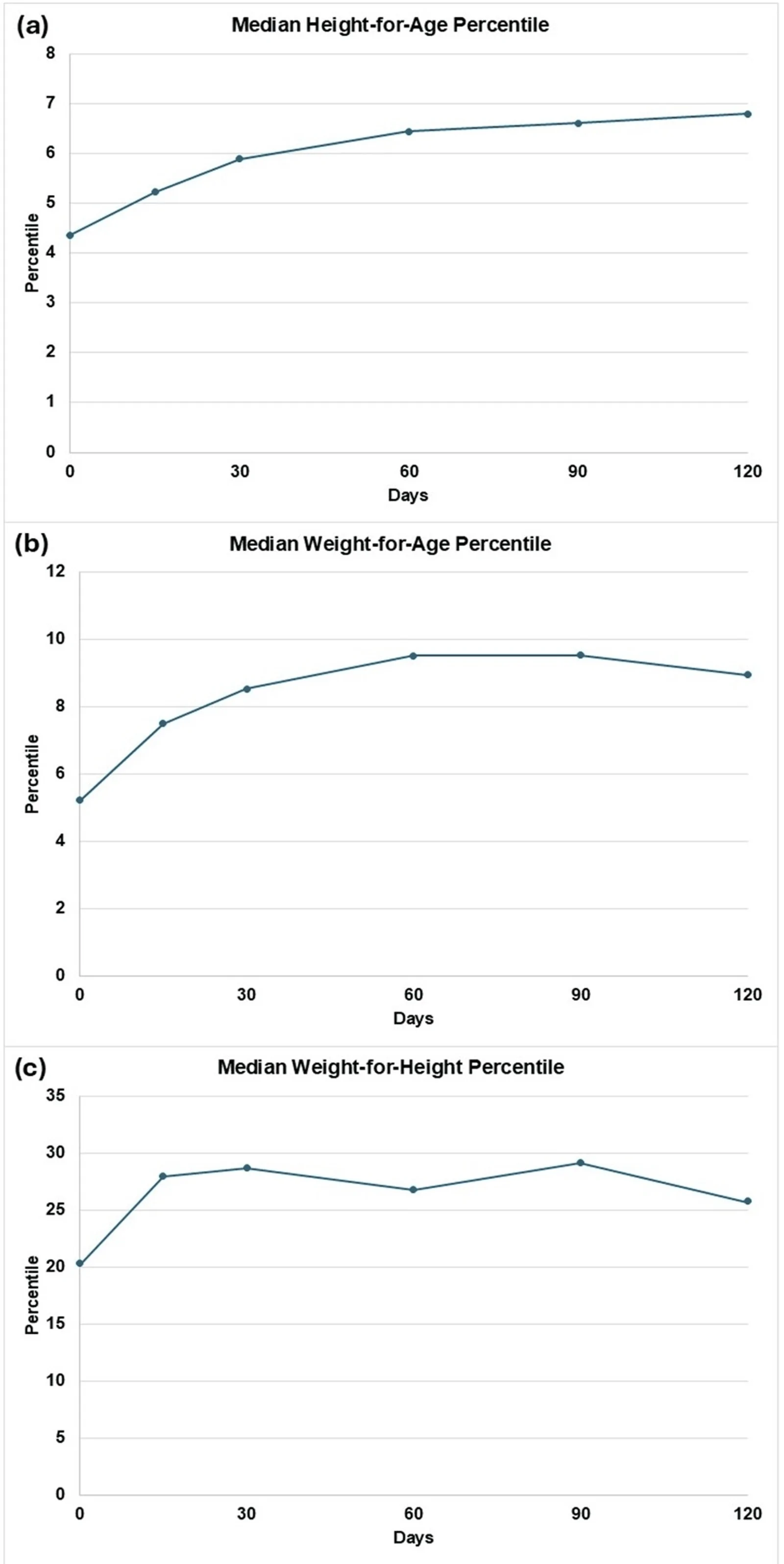
Changes in median anthropometric percentiles over 120 days in the intention-to-treat dataset. (a) Height-for-age; (b) Weight-for-age; (c) Weight-for-height.

The changes in median anthropometric percentiles over 120 days in the ITT dataset is presented in Figure 3.

**Figure 4:**
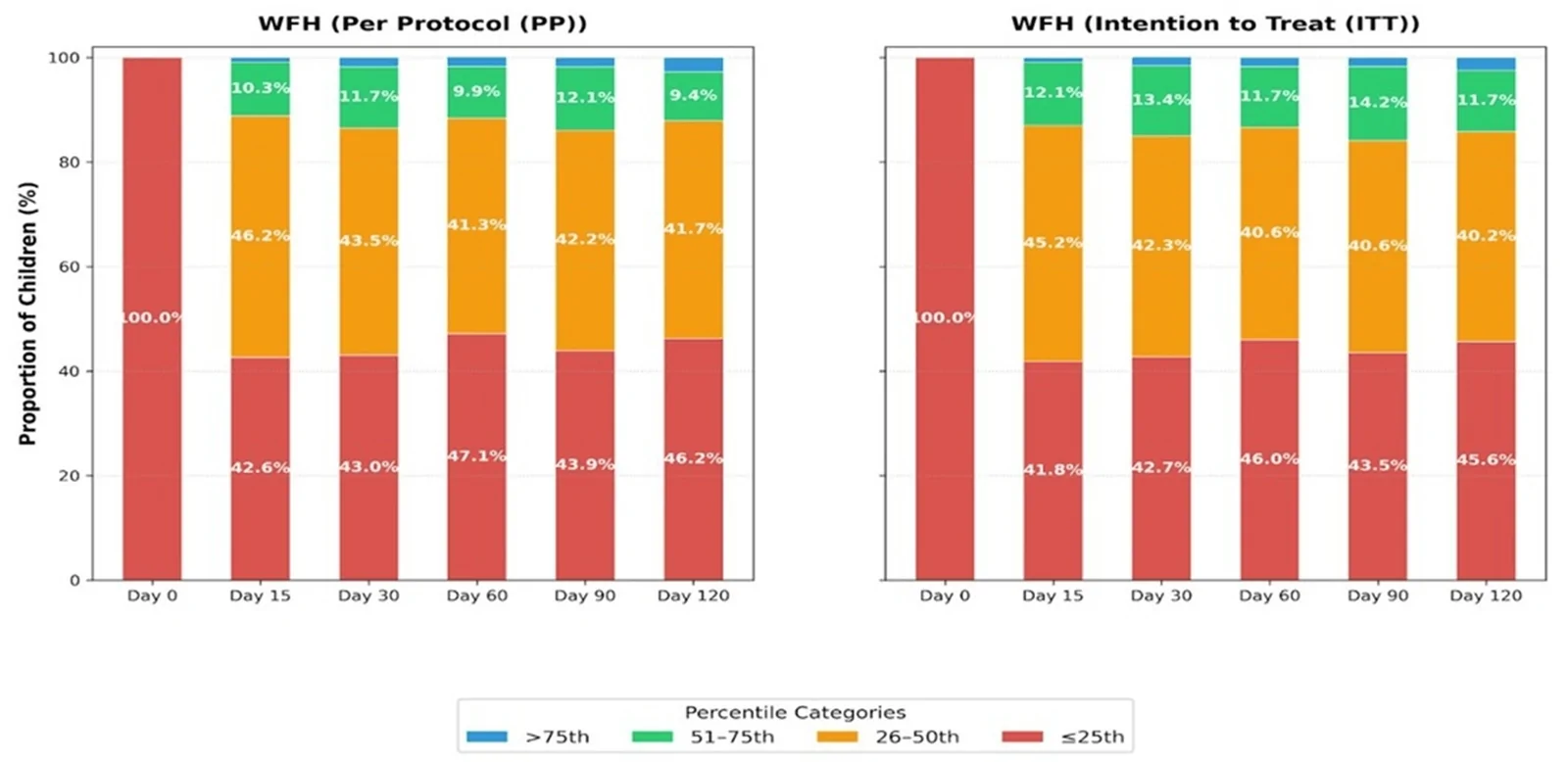
Percentile shift distribution over time for WFH in the PP and ITT datasets. Stacked bar charts show the distribution of children across WFH percentile categories at day 0, day 15, day 30, day 60, day 90, and day 120. The left panel presents the PP dataset, and the right panel presents the ITT dataset. ITT, intention-to-treat; PP, per protocol; WFH, weight-for-height

**Figure 5:**
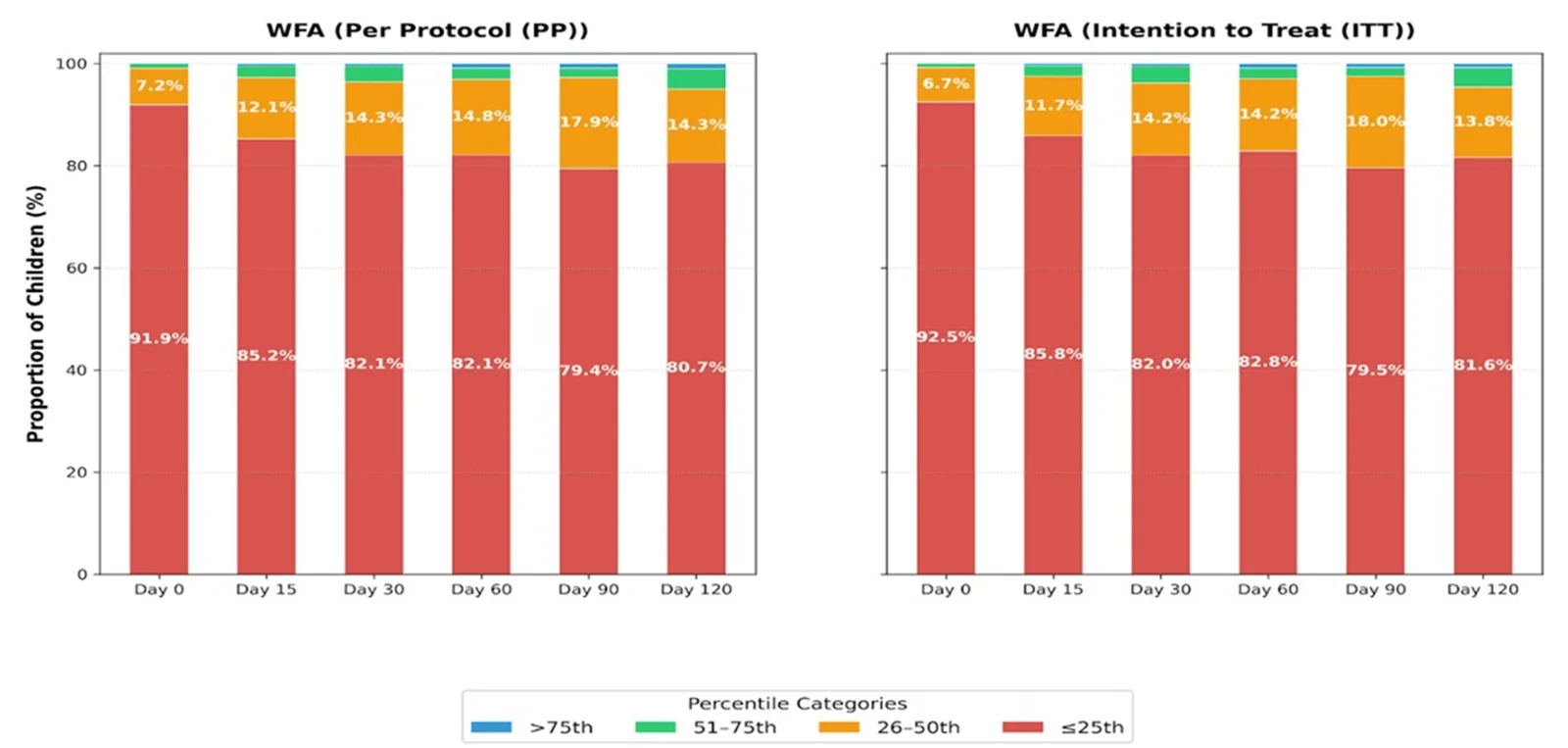
Percentile shift distribution over time for WFA in the PP and ITT datasets. Stacked bar charts show the distribution of children across WFA percentile categories at day 0, day 15, day 30, day 60, day 90, and day 120. The left panel presents the PP dataset, and the right panel presents the ITT dataset. ITT, intention-to-treat; PP, per protocol; WFA, weight-for-age

**Figure 6:**
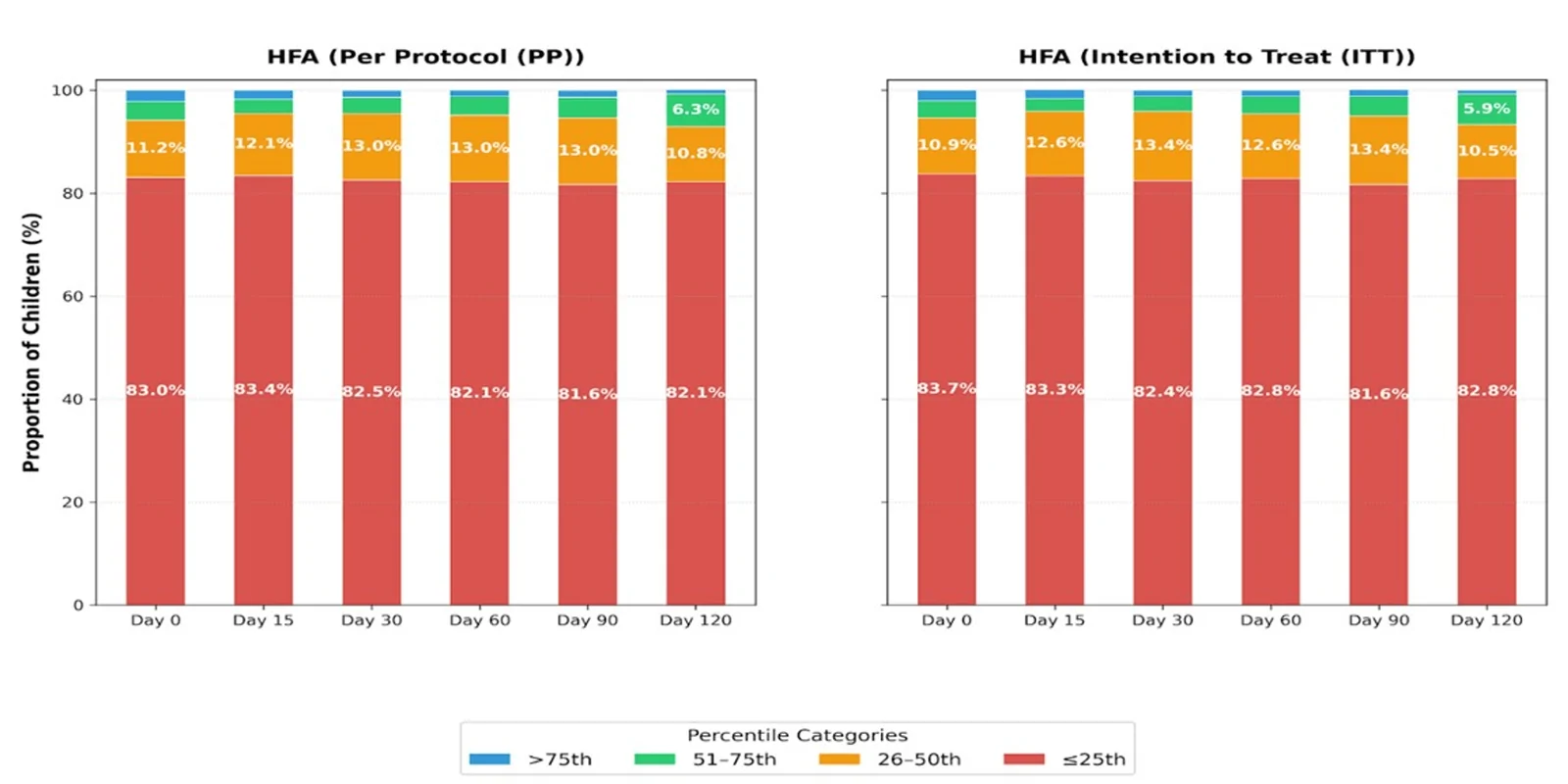
Percentile shift distribution over time for HFA in the PP and ITT datasets. Stacked bar charts show the distribution of children across HFA percentile categories at day 0, day 15, day 30, day 60, day 90, and day 120. The left panel presents the PP dataset, and the right panel presents the ITT dataset. HFA, height-for-age; ITT, intention-to-treat; PP, per protocol

The percentile-shift distributions showed consistent findings between the PP and ITT datasets, with the clearest redistribution observed for WFH, where the proportion of children at or below the 25th percentile decreased markedly after day 15 and remained lower through day 120; smaller shifts were observed for WFA and HFA (Figures 4-6).

## Discussion

This single-arm interventional study evaluated the impact of a nutrient-dense ONS administered with dietary counseling on growth, nutrient adequacy, and health outcomes in nutritionally at-risk Filipino preschool children. Over the 120-day intervention, significant improvements were observed in absolute weight and height and in age-standardized WFA, HFA, and WFH percentiles and z-scores. WFH responded rapidly, with the proportion of children at or below the 25th WFH percentile decreasing from 100% to 42.6% by day 15 and to 46.2% by day 120. WFA also improved progressively, with the proportion at or below the 25th percentile decreasing from 91.9% to 80.7%, whereas HFA changes were smaller, with the corresponding proportion decreasing from 83.0% to 82.1%. Improvements in growth parameters were observed from day 30 and remained significantly sustained through 120 days of study follow-up. The ITT sensitivity analysis showed a similar direction and magnitude of findings, suggesting that the growth outcomes were not materially altered by participant attrition or protocol non-completion. These findings align with previous trials in which ONS, particularly when combined with dietary counseling, improved weight-related indices and nutrient adequacy in children at nutrition risk [13,14,19,22-24].

Recent expert consensus emphasizes HFA as a critical indicator of linear growth and chronic undernutrition [17]. In our study, mean height increased from 95.5 cm to 98.5 cm, while the median HFA z-score shifted from -1.7 to -1.5 over 120 days. Although statistically significant, the magnitude of HFA change was modest and should be interpreted in the context of the chronic nature of linear growth faltering. This is notable given the mixed evidence on ONS effects on HFA: some RCTs reported greater height gains with ONS than with dietary counseling or usual diet alone, whereas others observed no significant impact [12-14,22-24]. Such discrepancies reflect differences in follow-up duration, baseline nutritional status, dose and duration of ONS, and supplement composition. Linear growth often lags behind weight gain and may only become evident after three or more months of adequate intake [25,26]; shorter studies or those using lower ONS doses (<224-448 kcal/day) may therefore underestimate height effects compared with longer or higher-dose interventions [12-14,22-24].

The temporal pattern of anthropometric change also warrants consideration. The longitudinal pattern suggested early anthropometric improvement followed by relative stabilization during later follow-up. This pattern may indicate that the largest short-term improvement occurred early after initiation of ONS and dietary counseling, followed by a stabilization phase as children moved toward a less nutritionally at-risk status. However, because the study was single-arm and was not designed to compare different treatment durations, the data should not be interpreted as establishing an optimal duration of supplementation. Rather, the findings support the feasibility and potential effectiveness of such a community-based intervention, with ongoing anthropometric monitoring to guide individualized continuation, transition, or discontinuation decisions.

Dietary counseling remains a key component of managing at-risk children, aiming to improve caregiver knowledge and feeding practices. However, its isolated impact on growth and nutrient adequacy is often modest and highly dependent on caregiver compliance and contextual factors such as socioeconomic constraints, food availability, and cultural practices [22,27-29]. Previous trials have shown that counseling alone frequently fails to achieve target nutrient adequacies in most children [22,28,29]. In contrast, evidence suggests that adding ONS to counseling yields greater improvements in energy intake and growth outcomes than counseling or a usual diet alone, with weight indicators responding within the first month and height-related gains emerging after approximately three months [30]. In the present study, in which counseling was concurrently delivered with ONS, the relative contribution of each component to growth and nutrition outcomes remains to be specifically assessed. Nevertheless, the findings seem to argue for a combined intervention in which the ONS addressed immediate nutrient gaps while counseling supported feeding practices, adherence, and maintenance of habitual food intake.

The composition of ONS is central to its effectiveness. Polymeric formulas providing a balanced mix of macronutrients and micronutrients have been shown to support growth in undernourished children [30]. An adequate protein/energy ratio is especially important; very low ratios, as seen when only fat or carbohydrate is added to feeds, can compromise lean mass accretion during catch-up growth [16,18]. A protein/energy ratio of about 8.9%-12% has been suggested as optimal for growth-faltering children [31]. The ONS used in this study was prescribed as two 250-kcal servings per day and provided a protein/energy ratio of 12%, together with fat, carbohydrate, calcium, iron, zinc, iodine, vitamins A, C, D, and B, and functional ingredients, including DHA, partially hydrolyzed guar gum, and *Lactobacillus rhamnosus* GG. This composition is consistent with evidence that micronutrient supplementation alone is often insufficient without adequate energy and macronutrient support [32-35]. Therefore, the present results reinforce the view that ONS, especially when combined with counseling, represents a comprehensive approach to addressing growth faltering more effectively than dietary counseling or micronutrient supplementation alone.

In this study, the EAR-based analysis suggested substantial reductions in nutrient inadequacy during follow-up. Energy inadequacy decreased from 76.0%-79.4% at baseline to 11.9%-15.6% at day 120, and protein inadequacy decreased from 24.0%-27.8% to 0.0%-0.8%. Calcium, iron, vitamin A, and vitamin B1 inadequacy also decreased substantially, whereas residual inadequacy persisted for zinc, fat, and vitamin C at day 120. These findings suggest that the combined intervention helped close several major nutrient gaps but did not fully normalize all dietary inadequacies. Dietary diversity also improved, but because DDS was adapted for children aged three to five years, it should be interpreted as a descriptive indicator of dietary variety rather than as a validated diagnostic measure of micronutrient adequacy. Similar patterns have been reported in other studies, where children receiving ONS achieved greater gains in energy intake, weight, and height than those given counseling alone, a placebo, or a usual diet [29,30,36]. 

The adherence and safety findings support the feasibility of this community-based intervention. VOT-based compliance ranged from 92.5% to 98.3% of prescribed doses, and returned-tin accountability was complete, suggesting that the prescribed two-serving daily regimen was feasible in this setting. This finding was consistent with previous pediatric ONS evidence, in which high prescribed-dose compliance was reported in children receiving ONS, including a systematic review reporting mean compliance of 98% and a recent randomized trial reporting median compliance of 95.9% at 120 days [22,37]. Although VOT has been studied mainly in tuberculosis treatment, smartphone-enabled video observation has been shown to improve treatment observation and to provide an acceptable, scalable method for monitoring daily or multiple daily dosing [38]. Upper respiratory infections and diarrhea were recorded during follow-up, but all adverse events were mild to moderate unless hospitalization was required; all four serious adverse events were judged unrelated to ONS, no dose modification or discontinuation was required, and all affected participants recovered. These observations were consistent with a recent pediatric ONS randomized trial in which adverse events were mostly mild, gastrointestinal-tolerance events were infrequent, and serious adverse events were not considered related to the intervention [22]. Because the study was conducted without a control group and during routine community exposure, these health events should be interpreted descriptively rather than as evidence of a potential effect of the intervention. This aspect needs to be explored in a dedicated and properly designed study.

To our knowledge, few studies have evaluated the impact of energy-dense ONS on growth and health outcomes in nutritionally at-risk preschool children. Key strengths of this study include the random selection of participating barangays, a relatively large sample size, VOT-supported adherence monitoring, product accountability, and the longitudinal design with multiple assessments over 120 days, which enabled detailed evaluation of changes in anthropometric indices, including z-scores and linear growth. The real-world, community-based setting further supports the practical applicability of this intervention at a population level. Limitations include the single-arm design without a control group, limiting control of confounding factors and the ability to definitively attribute all observed changes to the ONS. Baseline characteristics were not formally compared between completers and non-completers, although ITT sensitivity analyses were performed. Reliance on caregiver-reported dietary data introduces potential recall and social desirability bias. DDS was adapted for this preschool-aged cohort and should be interpreted as exploratory. Finally, the random selection of participating barangays may have improved the representativeness of the sampled urban area; however, it does not establish the generalizability or population-level effectiveness of the intervention.

## Conclusions

In conclusion, nutrient-dense ONS combined with dietary counseling was associated with improvements in the growth of nutritionally at-risk preschool children over 120 days. A measurable early growth response was observed by day 30, and improvements were sustained through the end of follow-up. Children exhibited significant improvements in age-standardized weight and height percentiles, growth velocity, energy and macronutrient intake, and adequacy. These findings support the potential value of ONS combined with dietary counseling as an effective strategy for addressing growth faltering and nutrient deficiencies in this vulnerable population. Further controlled trials are needed to confirm our findings and evaluate the long-term impact of these benefits on child development.
